# Clinicians' Views of Patient-initiated Follow-up in Head and Neck Cancer: a Qualitative Study to Inform the PETNECK2 Trial

**DOI:** 10.1016/j.clon.2021.11.010

**Published:** 2022-04

**Authors:** A. Lorenc, M. Wells, T. Fulton-Lieuw, P. Nankivell, H. Mehanna, M. Jepson, PETNECK2 Research Team, A. Karwath, B. Main, C. Firth, C. Gaunt, C. Greaves, D. Moore, E. Watson, G. Gkoutos, G. Ozakinci, J. Wolstenholme, J. Dretzke, J. Brett, J. Duda, L. Matheson, L.-R. Cherrill, M. Calvert, P. Kiely, P. Gaunt, S. Chernbumroong, S. Mittal, S. Thomas, S. Winter, W. Wong

**Affiliations:** § Institute of Cancer and Genomic Sciences, University of Birmingham, UK; ¶ University Hospitals Bristol and Weston NHS Trust, UK; ‖ Cancer Clinical Trials Unit, University of Birmingham, UK; ∗∗ School of Sport, Exercise and Rehabilitation Sciences, University of Birmingham, UK; †† Institute of Applied Health Research, University of Birmingham, UK; ‡‡ Oxford School of Nursing and Midwifery, Oxford Brookes University, UK; §§ Health Psychology Research Group, University of Stirling, UK; ¶¶ Nuffield Department of Population Health, University of Oxford, UK; ‖‖ Department of Midwifery, Community and Public Health, Oxford Brookes University, UK; ∗∗∗ Oxford Institute of Nursing, Midwifery and Allied Health Research, Oxford Brookes University, UK; ††† University Hospitals Bristol and Weston NHS Foundation Trust, UK; ‡‡‡ Institute of Head and Neck Studies and Education, Institute of Cancer and Genomic Sciences, UK; §§§ Bristol Dental School, University of Bristol, UK; ¶¶¶ Nuffield Department of Surgical Sciences, University of Oxford, UK; ‖‖‖ East and North Hertfordshire NHS Trust, Mount Vernon Cancer Centre, UK; ∗ QuinteT Research Group, Bristol Medical School, University of Bristol, Bristol, UK; † Nursing Directorate, Imperial College Healthcare, NHS Trust / Department of Surgery and Oncology, Imperial College, London, London, UK; ‡ Institute of Head and Neck Studies and Education (InHANSE), Institute of Cancer and Genomic Sciences, University of Birmingham, Birmingham, UK; § University Hospitals, Birmingham NHS Foundation Trust, Birmingham, UK

**Keywords:** PIFU, Patient-initiated follow-up, HNC, Head and neck cancer, Head and neck cancer, health care professionals, patient-initiated follow-up, qualitative, survivorship

## Abstract

**Aims:**

Current follow-up for head and neck cancer (HNC) is ineffective, expensive and fails to address patients' needs. The PETNECK2 trial will compare a new model of patient-initiated follow-up (PIFU) with routine scheduled follow-up. This article reports UK clinicians' views about HNC follow-up and PIFU, to inform the trial design.

**Materials and methods:**

Online focus groups with surgeons (ear, nose and throat/maxillofacial), oncologists, clinical nurse specialists and allied health professionals. Clinicians were recruited from professional bodies, mailing lists and personal contacts. Focus groups explored views on current follow-up and acceptability of the proposed PIFU intervention and randomised controlled trial design (presented by the study co-chief investigator), preferences, margins of equipoise, potential organisational barriers and thoughts about the content and format of PIFU. Data were interpreted using inductive thematic analysis.

**Results:**

Eight focus groups with 34 clinicians were conducted. Clinicians highlighted already known limitations with HNC follow-up – lack of flexibility to address the wide-ranging needs of HNC patients, expense and lack of evidence – and agreed that follow-up needs to change. They were enthusiastic about the PETNECK2 trial to develop and evaluate PIFU but had concerns that PIFU may not suit disengaged patients and may aggravate patient anxiety/fear of recurrence and delay detection of recurrence. Anticipated issues with implementation included ensuring a reliable route back to clinic and workload burden on nurses and allied health professionals.

**Conclusions:**

Clinicians supported the evaluation of PIFU but voiced concerns about barriers to help-seeking. An emphasis on patient engagement, psychosocial issues, symptom reporting and reliable, quick routes back to clinic will be important. Certain patient groups may be less suited to PIFU, which will be evaluated in the trial. Early, meaningful, ongoing engagement with clinical teams and managers around the trial rationale and recruitment process will be important to discourage selective recruitment and address risk-averse behaviour and potential workload burden.

## Highlights

• Head and neck cancer clinicians agree follow-up regimens need to change.
• If proven effective, patient-initiated follow-up may improve patient-centredness.
• Clinician concerns include the suitability of patient-initiated follow-up for all.
• Patient-initiated follow-up may impose additional burden on some clinicians.
• Patient-initiated follow-up may not suit ‘disengaged’/high-risk/deprived patients.

## Introduction

There are over 12 000 new cases of head and neck cancer (HNC) in the UK annually [1]. The incidence of oropharyngeal cancer is rising, largely driven by human papillomavirus (HPV) infection [2]. HPV-related cancers affect younger patients and have better prognoses than HNC caused by tobacco/alcohol, so the cohort of HNC survivors is growing rapidly. UK national HNC guidelines recommend follow-up appointments every 2 months for the first 2 years after treatment, and then every 3–6 months for the next 3 years [3].

Current HNC follow-up has some limitations. First, follow-up often fails to meet patient needs, including information, holistic, survivorship and psychosocial support needs [[4], [5], [6], [7]]. HNC patients have particularly diverse needs, due to extensive comorbidities and functional disruption [6,8], not accounted for by the ‘blanket approach’ to follow-up care [6,9]. Second, current follow-up regimens may not be the most effective or cost-effective at detecting cancer recurrence [[10], [11], [12], [13]], nor provide a survival advantage [14]. Third, routine scheduled appointments are resource-intensive and potentially unsustainable given increasing HNC incidence [1]. Finally, some HNC patients feel that follow-up is too frequent and would welcome less-intensive schedules [15,16].

Alternative follow-up paradigms, including those used in other cancers, therefore need to be considered and researched for HNC [10,12,16,17]. Systematic reviews suggest that patient-directed surveillance or patient-initiated follow-up (referred to here as PIFU) may have comparable clinical outcomes to and better patient satisfaction and cost-savings than regular follow-up, in a range of conditions [18]. In cancer, findings vary – clinical outcomes of PIFU compare to regular follow-up in prostate cancer [19], colorectal cancer [20] and breast cancer [21], but a review (various cancers) found little difference in quality of life or disease recurrence [22]. In breast cancer in the UK, PIFU/open access nurse-led clinics provide a feasible alternative to regular appointment-based follow-up [23,24].

Patients may prefer self-management approaches – as found for endometrial [25], breast [26], colorectal [20] and prostate cancer [19]. In HNC, there is limited, poor-quality, retrospective and conflicting evidence regarding the relative efficacy of PIFU to regular follow-up – studies show no overall survival difference [[27], [28], [29]] or better survival in routine follow-up [30]. In prospective studies, recurrence was more commonly identified via patient-identified symptoms than at appointments [29,[31], [32], [33]] and in a feasibility study of ‘enhanced’ follow-up (patient education and encouragement to contact the clinic) patients did make contact regarding ‘red flag’ symptoms [34]. Risk-stratified follow-up is also recommended in HNC [5,9,13,17,35], e.g. de-intensified follow-up for lower risk of recurrence [13,36,37].

Data on HNC clinicians' views of follow-up are limited, but suggest that they feel that HNC survivors are neglected and have complex, enduring and unaddressed post-treatment needs [7]. Clinicians broadly support a patient self-management approach in HNC [38,39], although unlike other cancers, alternative models of follow-up in HNC have been slow to develop and many clinicians have been nervous of change [[40], [41], [42]].

PETNECK2 is a programme of research (NIHR200861) with an embedded randomised controlled trial (RCT) designed to determine whether PIFU is more effective than regular follow-up for HNC. PETNECK2 builds on the successful results of the first PETNECK study, where a 3-month positron emission tomography-computed tomography (PET-CT) scan reliably detected patients requiring neck dissection, avoiding unnecessary surgery for those at low risk of recurrence, reducing harm and costs [43]. The PETNECK2 trial will randomise eligible patients to PIFU or standard care (regular scheduled follow-up appointments).

As implementing PIFU in HNC is innovative and may be challenging, preliminary research with clinicians and patients explored the feasibility, barriers and concerns [44]. Qualitative work prior to an RCT can be invaluable in informing study design, especially for new interventions [45,46]. This article reports the initial research exploring clinicians' views about HNC follow-up and PIFU, including concerns and barriers, prior to the PETNECK2 trial.

## Materials and Methods

In the UK, current HNC follow-up consists of regular scheduled appointments with multidisciplinary HNC teams [3] and usually a system for patients to contact the clinic in between appointments [12]. In PIFU, patients will receive standard follow-up for the first year post-treatment. Then, at study entry, they will have a PET-CT scan. If this scan is negative, they will have PIFU instead of regular scheduled clinic follow-up appointments. In addition to the PET-CT scan, patients will receive an allied health professional (AHP)/nurse-led education session, an information and support resource (app, website or paper) and rapid access to urgent clinical appointments within 2 weeks. The information and support resource provides information on symptoms to be aware of, a diary to record/monitor symptoms and contact details for easy access to their clinical team. It also includes information on patient concerns, patient/caregiver support, living well and peer support groups.

Online (Microsoft Teams) focus groups were conducted with ear, nose and throat and maxillofacial surgeons, oncologists, clinical nurse specialists (CNSs) and AHPs (speech and language therapists [SLTs], dietitians and radiographers), facilitated by AL and MJ, audio-recorded and transcribed verbatim. Non-verbal behaviour was noted during focus group to supplement/clarify transcripts. Participants joined from their home or clinic setting. The facilitators did not know the participants beforehand, but some participants knew each other or were colleagues. Participants in each focus group were the same profession, although CNSs and AHPs were grouped together.

Clinicians were recruited via personal contacts of the team and multidisciplinary professional body mailing lists representing HNC clinicians (British Association of Head and Neck Oncologists [BAHNO] and British Association of Head and Neck Oncology Nurses [BAHNON]). Some participants suggested colleagues. Individuals were given written information, invited via e-mail or face-to-face and followed up. All provided written consent prior to taking part.

During focus groups, clinicians were asked for details of and their views on current follow-up care at their centre, the co-chief investigator outlined the PETNECK2 study, then we asked about the acceptability of the proposed intervention and the RCT design, their preferences, margins of equipoise and potential organisational barriers and ways of overcoming them. Views on the content and format of PIFU were also explored.

Data were interpreted using inductive thematic analysis. A. Lorenc carried out all of the data analysis, with M. Jepson checking themes and subthemes. Nvivo software (version 20.5.1.940) was used to facilitate data analysis. Data were examined for credibility, context, language, negative cases and rival explanations. Inductive themes not directly related to questions or prompts were emphasised in the results.

A patient advisory group (PAG) was convened for the programme and met regularly. The PAG provided valuable input into the study design, including recruitment methods and interview topic guides. PAG representatives attended meetings and provided feedback on the results.

The study was approved by the North East Tyne & Wear South Research Ethics Committee, the Health Research Authority and Health and Care Research Wales (reference 20/NE/0102). All names of people, places and any other identifiable information were removed from transcripts.

## Results

Of 39 clinicians agreeing to take part, five could not attend due to illness or clinical obligations, giving a sample of 34 clinicians from 13 different HNC centres (between one and six clinicians from each centre), in England (including six of the seven NHS England regional teams), Wales and Scotland: 10 oncologists, 10 surgeons (five ear, nose and throat, five maxillofacial), eight CNSs, four SLTs, one dietitian and one research radiographer. Most worked in large cancer centres, with some from smaller hospitals. Eight focus groups were held – three with surgeons, two with oncologists, two with AHPs and CNSs and one with CNSs only – and one individual interview with a CNS.

We generated six main themes, discussed below, with tables of illustrative quotes for each. The first three themes had the most data: belief in the need for change; the inflexible, paternalistic and unresponsive nature of follow-up as unsuitable for a diverse clinical population; and the perceived unsuitability of PIFU for disengaged patients. Themes that came up less frequently and seemed less important were: concerns that PIFU would worsen patient anxiety and not address psychosocial needs; PIFU resulting in delayed detection of recurrence; and challenges of implementation into services.

### Change is Needed

Clinicians agreed that the current HNC follow-up regimen is inadequate and needs to change, with enthusiasm for the PETNECK2 trial to develop and evaluate innovative follow-up. The key limitations of current follow-up were rigidity and unresponsiveness to patient need, lack of evidence and effectiveness, strain on resources and patient anxiety. Quotes on this theme can be found in Table 1.

**Table 1:** Quotes on the need for change

Limitations of current follow-up	It's prescriptive and certainly not evidence-based … it's a little bit archaic, and I think, for a long time, many of my colleagues have felt that we could look at a more sensible way of following up patients, and certainly more evidence-based. And I think this is very, very timely (001, surgeon) It's [follow-up] very random and I still haven't managed to find anything that is concrete and scientifically proven. But I think you go with the flow to say 4–6 weeks and guided by the factors that I've just told you [patient education, risk of recurrence/metastasis, chance of treatment success] (056, surgeon) I think those of us with high [patient] volumes, which would be most of the people involved in the trial, I imagine, would be more open [to change] because they recognise the challenges of clinics full of patients (020, surgeon) Very often they'll come for an appointment because that's their designated time, but then a couple of weeks later, 3 weeks later, you'll get a phone call and they'll say either they've noticed something new, or they had it, but they didn't want to mention it because of other things, or they forgot, and this, that and the other (006, speech and language therapist)
Elements of patient-initiated follow-up in existing follow-up	[follow-up is] two monthly in the first year, three monthly in the second year follow on. I've actually had to, sort of, push that out a little bit with some of the patients … if I think that they're better prognosis (005, oncologist) [patient education sessions] to reflect and debrief and move forward with their lives after their treatment, and that's been a full day of hospital site where there's been a lot of supportive interaction. It's quite labour intensive to set up from our point of view but the outcomes of it have been very positive (004, speech and language therapist)
COVID-19 accelerating change	We were looking at trying to do a telephone follow-up [and] full survivorship package … that's been ongoing probably actually on and off for about 6 years, but it's really stepped up now actually, with COVID (078, clinical nurse specialist)
Resistance to change	There will be some people [clinicians], I think, that … the way they approach risk, or just their attitude, they may just say, ‘Well, no, I'm … not willing to engage in that [patient-initiated follow-up]’ (017, surgeon) It's always a new challenge, the inertia of the machine that's steadily rolling forward. We all, often, are trained in different parts of the country in the same model, and trying to introduce change in that front in some units can be a challenge (020, surgeon)

Many clinicians and services had either already amended their follow-up or aspired to, demonstrating a willingness to change. Changes included informal tailoring of protocols for specific patients and formal changes including elements of PIFU, such as reduced frequency of appointments and patient education sessions/materials, e.g. health and wellbeing events. Being able to call a CNS in between follow-up clinic appointments was accepted as ‘normal practice’.

COVID-19 pandemic restrictions had accelerated and provided opportunities for change (mainly telephone/virtual consultations replacing face-to-face and reduced appointment frequency), boosting clinician, service and patient enthusiasm for change.

However, despite enthusiasm, clinicians suggested some colleagues may be reluctant to change, due to being risk-averse or because of inertia within services. Suggested solutions were to explain the study rationale and obtain high-level strategic buy-in to reassure clinicians of the study's importance.

### Follow-up is Inflexible, Paternalistic and Unresponsive to Need

Clinicians discussed current follow-up being rigid and unresponsive to patient need and felt that flexibility and adaptability were particularly necessary in HNC due to the wide range of causes, severity, outcomes and prognoses. Quotes on this theme can be found in Table 2.

**Table 2:** Quotes on follow-up being inflexible, paternalistic and unresponsive to need

Need for flexibility	[we need follow-up on a] more intelligent basis rather than the, the blunt tool of everyone gets exactly the same thing (020, surgeon) I think we're probably all in agreement that there is room for improvement in the way that we see the patients on their follow-up protocol. It sounds like we've all got a very similar, traditional one-size-fits-all approach to our follow-up (027, oncologist)
Responsive to need	You can bounce people back a few months if they're well, and I think we feel comfortable with that to some extent, but then get a little bit unsure of how much to keep bouncing them back (003, surgeon)
Patient-initiated follow-up being patient-centred	I think it's [patient-initiated follow-up] kind of giving them that ownership back because we've taken it for so many months doing the treatment that we need to just give it back again (043, clinical nurse specialist)
Risk-stratification	Patients who've been able to quit smoking or alcohol use, or semi reduce it significantly, might be at low risk of recurrence, and perhaps those are patients who could be on a less stringent follow-up. So, I don't know if you are going to stratify according to risk factors as well (062, surgeon)
PET-CT scan concerns	If a PET is not sensitive to pick that [recurrence] up, and I don't think it is, then where do those patients sit, patients who, potentially, have field change, who have multiple malignancies that arise within field change? Should they be excluded, or where would they fit in this pathway? (028, surgeon)

PET-CT, positron emission tomography-computed tomography.

Some clinical teams had already made informal changes to follow-up, as mentioned above, mostly responding to a patient's level of risk or need and clinical factors. Changes appeared un-protocolised and undocumented – some identified a need for more clarity and guidance. Changes were mostly reduced appointment frequency, but also early discharge (<5 years) or different clinicians providing follow-up.

Some clinicians felt that the current system is paternalistic, not adequately giving patients control or addressing long-term effects or patient needs, which are better addressed by holistic needs assessments. Some, mainly AHPs and nurses, felt that PIFU might be more patient-centred and empowering, restoring patient autonomy and ownership over their bodies. A few commented that this may reduce patient anxiety.

There was debate around the adaptability of PIFU and suitability for patients at higher risk of recurrence, which are discussed below.

There was also concern that the PET-CT scan may not detect recurrence in certain types of HNC.

#### Concern about Disengaged Patients

The most common concern about PIFU was perceived unsuitability for patients seen as ‘disengaged’. Quotes on this theme can be found in Table 3. This group of ‘traditional’ HNC patients were reported to be at higher risk of cancer recurrence, engaged in high-risk behaviour, experiencing other mental/physical health issues and vulnerabilities, and possibly of lower socioeconomic status/education level (although one SLT warned against demographic generalisations). Clinicians were concerned about poor engagement in care and possibly low risk awareness, making patients less ‘able to deal with’ PIFU (017, surgeon). It was assumed that they would be unlikely to initiate contact during PIFU and were adversely affected by reduced clinical contact, potentially delaying presentation of recurrence/metastasis and worsening outcomes.

**Table 3:** Quotes on disengaged patients

Disengagement in certain groups	I keep banging on about, you know, our complex group of patients, because they are. But we do have one end of the scale with the old fashioned, typical head and neck patient that prop the bar up and smoked countless amounts of cigarettes, to the HPV typical patient that we're seeing now (064, clinical nurse specialist) A lot of the head and neck cancer patients … the traditional smoker, drinkers, we struggle to get them to come for follow-up. So, I think we've got to be careful about recruiting these patients (011, oncologist) How well they're [patients] educated is an important factor. They are likely to pick up the cancer much sooner if they notice any change compared to somebody who is poor socioeconomic [status] (056, surgeon) A small group of [lower socioeconomic status] patients … will say ‘just do what you think is right’. They don't want to know, you know? I would not trust them, not because I don't like them, it's just that I can't trust them to make a sensible decision to come back if they have a concern (056, surgeon) We have some patients that don't come to any follow-ups. I think those patients, because of their sociological backgrounds, they don't engage with health (023, speech and language therapist)
Inclusion in PETNECK2 trial	Somebody who struggles to attend an appointment or finds themselves in the pub very first thing in the morning is not the candidate [for this trial] (056, surgeon) The type of people who you get, who will engage with the trial, are the kind of people who are taking a bit more responsibility for their own health and engaging in health. And so what you find from your trial might be completely different to the clinical head and neck picture (008, speech and language therapist)

HPV, human papillomavirus.

There was also concern about clinicians not approaching this group for the PETNECK2 trial or patients refusing participation. Participants debated whether they should exclude or actively recruit them to improve generalisability. One proposed solution was flexible PIFU with optional extra support (some clinician appointments).

#### Patient Anxiety and Need for Reassurance

Although regular follow-up appointments were acknowledged to cause some patient anxiety (by exacerbating fear of recurrence), many clinicians (especially CNSs) perceived that patients found appointments reassuring and were concerned how PIFU would address this, although the additional PET-CT scan may reassure them (see Table 4). An additional concern was the patient/carer burden of PIFU. One AHP suggested carefully considering patients' psychosocial needs, including fear of recurrence. Others advised managing patient expectations regarding changing follow-up.

**Table 4:** Quotes on final themes

Patient anxiety and need for reassurance	You do always get that group of patients that want to come in and feel reassured just by it, it sounds crazy but just by having the doctor's hands on their neck and things like that they basically feel reassured (036, clinical nurse specialist) I think that most of our patients actually don't want that [telephone follow-up]. They want, they want to be seen. They want to come in (078, clinical nurse specialist) [Patient-initiated follow-up] is relying on a sense of responsibility and learnt experience of a patient, which is empowering long term, but requires biopsychosocial interventions for patients because actually just teaching a method of ‘these are your risk factors. This is where you need to get in touch with us’ has the potential to raise people's anxieties without empowering them significantly enough that they can manage this without a burden of responsibility, and for their carers as well (023, speech and language therapist)
Detecting symptoms	[patients may not attend clinic] because they're holding back a problem or they're scared. And it's really how those things get identified, because this potentially can be the way that people keep a problem [hidden] that we would have seen by looking in the whites of their eyes (013, oncologist)
Issues with implementation into services	I think the main concern was if it [patient-initiated follow-up] would add to the workload (021, clinical nurse specialist) Speech and language services and clinical nurse specialist services are historically underfunded, and dietetic services, so actually our ability to engage in this in a meaningful way and get backfill and all the rest of it, that might be difficult on a practical level. But I think conceptually people would be into it, but I think it is the practical facets that would be tough (023, speech and language therapist)

#### Detecting Symptoms

Some clinicians were concerned that PIFU would impede recurrence/metastasis detection, based on delayed presentation (from patients' fear of recurrence and ‘ignoring’ symptoms), patients failing to recognise or detect symptoms or lack of face-to-face consultations and examinations (see Table 4). Detailed and specific information on important symptoms was recommended, but also avoiding overburdening patients/carers.

#### Issues with Implementation into Services

Regarding implementing PIFU into National Health Service care, two themes were discussed, mostly by CNSs/AHPs (see Table 4). First, the route to urgent appointments is important and needs to be clear, efficient, reliable and quick, possibly with multiple contacts, although CNSs were preferred due to specialist clinical knowledge, approachability and regular patient contact. The second theme was concern around staffing and potential additional nursing workload.

## Discussion

UK clinicians appear keen to change HNC follow-up due to limitations in its evidence base, effectiveness, resource efficiency and flexibility. Existing service changes were accelerated by COVID-19 restrictions. Clinicians saw the paternalistic and unresponsive nature of follow-up as unsuited to the diverse clinical population and PIFU as potentially more patient-centred. However, some were concerned that certain patients (higher risk of recurrence and so-called ‘disengaged’ individuals, possibly engaging in high-risk behaviours and with lower socioeconomic status/education) may not initiate contact during PIFU, worsening outcomes. Others were concerned that PIFU would increase patient anxiety, not meet psychosocial needs and delay recurrence detection. Potential implementation issues included resistance to change, a clear and reliable urgent appointment system and burden for already overworked nurses and AHPs. The value of qualitative work prior to an RCT was clear, with crucial issues – and potential solutions – identified, e.g. patient inclusion criteria, the need for tailored interventions for certain patient groups and barriers to change/implementation.

As previously identified [47], HNC follow-up regimens appeared largely consistent between centres, with some local adaptations (as recommended [3]). Some elements of PIFU mentioned have been piloted in HNC – patient education interventions [34] and holistic appointments [48]. Patients calling between appointments is established in follow-up and is utilised by around 8% of UK patients [12]. Clinicians perceived already known limitations with HNC follow-up, such as lack of flexibility to address the wide-ranging needs of HNC patients [6,9], expense [49] and lack of evidence [[10], [11], [12], [13],^17^] and seemed willing to change their provision. One UK HNC service is planning to change their follow-up to a patient-centred approach for low-risk patients [37]. However, despite a need for and a willingness to change, some clinicians anticipated resistance to change among colleagues/services, a known barrier for HNC [40] and for implementation of PIFU in other conditions [44].

Although clinicians had some concerns about patients recognising symptoms, patient-identified symptoms may detect HNC recurrence as often as appointments [29,[31], [32], [33]]. However, concerns about delayed help-seeking may be valid, as studies suggest that patients may not request urgent appointments despite recognising symptoms [33,50]. Help-seeking may be hindered by the physical, emotional and social disruption from HNC [51] and barriers to patient self-management [52]. HNC patients may feel they are ineligible for treatment and understate concerns, perhaps due to a sense of diminished self from functional, social and existential losses and low self-esteem [51] or feeling judged for their health behaviours, as in lung cancer [53]. Physical issues, including challenges with travelling, may restrict engagement with services [54]. These barriers may lead to hesitancy coming forward with concerns during PIFU.

Clinicians were particularly concerned about some patient groups not initiating contact. Concerns about ‘traditional’ HNC patients who smoke or drink maybe be justified. Although many HNC patients do quit smoking, a significant minority of patients with lower income or educational levels continue to smoke and drink alcohol [54] and are less likely to seek help [55] or engage with services [56,57], leading to potential recurrence [58]. Engagement with services, including PIFU [25], may be limited by a lack of education and income, living alone and being out of work [53,58,59]. Concerns about the higher risk in this group may also be justified, as poorer survival is associated with deprivation, low income and lower educational level [60].

Clinicians, especially CNSs, worried that patients would miss the reassurance of regular follow-up, leading to anxiety. Fear of recurrence is prevalent in HNC [61] and it concerns clinicians [7] and can influence follow-up preferences [16]. However, despite regular follow-up being reassuring [4], it inadequately addresses patients' holistic psychosocial needs [[5], [6], [7]], including in HPV-related HNC despite lower risks [62]. PIFU may better address these needs [19] and give patients more control over their lives and health [63]. However, clinicians will need to manage patient expectations, as patients will probably expect extensive testing and intensive follow-up visits [64].

This study has highlighted the burden upon nurses/AHPs in HNC services – despite their importance for patient experience, leadership, quality of care, safety and productivity and efficiency [65], and guideline recommendations for them to see all patients [66], this is often not achieved (present at 23% of existing clinics [12]) and they struggle to meet rehabilitation and survivorship needs. There is a shortage of cancer CNSs and dedicated HNC AHPs [67] and their confidence in some areas may be low [5]. Nurse/AHP workload burden from PIFU is therefore a concern, especially in HNC, where care is particularly demanding and time consuming [68]. Although workload and capacity issues are known barriers in implementing PIFU [44], cost-effectiveness studies are needed to evaluate the cost implications [18]. Undertaking this qualitative work enabled anticipation of potential concerns to be addressed during trial set-up. Consequently, PIFU for the PETNECK2 RCT will be carefully designed and implemented via workshops with clinicians and patients.

### Implications

This study highlights the need for and the willingness of the HNC community to consider alternative follow-up protocols [12] and justification for the PETNECK2 study. Our results highlight that clinicians feel that HNC patients have unmet psychosocial needs during follow-up.

The PIFU intervention will be carefully designed to address concerns, including ensuring patients feel confident to contact clinics, promoting and facilitating self-management, providing information and education on ‘red flag’ symptoms, providing clear robust referral routes and reassuring patients that their concerns are legitimate [12,25,33,50,52]. The intervention development process purposively includes patients from less engaged groups and uses tailored strategies for communication issues. However, it is likely that some patients/groups of patients will be unwilling to participate in the trial or not engage during PIFU, data which will be collected as part of the PETNECK2 trial.

Acknowledging the psychosocial impact of HNC and fear of recurrence is important [51]. PIFU will use strategies to avoid aggravating patients' psychosocial issues and promote psychological well-being and coping skills [7,69]. It is hoped that PIFU will beneficially impact mental wellbeing by improving self-management and addressing holistic needs. In addition, the PIFU information and support resource will contain specific information and advice on mental wellbeing, and patients with substantial psychological issues will be referred to mental health services as per usual practice. Participating clinicians will be asked to introduce the possibility of PIFU at an early stage to avoid expectations of regular medical appointments, as patient expectations are important in the patient experience [64].

Clinicians clearly have reservations about the effectiveness of PIFU, at least for some patient groups. The PETNECK2 trial will engage early with trial sites and clinicians to emphasise the trial rationale and help build confidence that PIFU will not be inferior to current standard follow-up regimens [39]. Local managers and teams will be engaged with, before and during trial set-up, through team and individual meetings to discuss any concerns, barriers and issues, and pre-recorded videos and a guide to implementation. It is also important to consider contextual issues at departmental levels, such as empowered leadership and team members, trust in colleagues and patients, and capacity to make changes, that are likely to impact the progress of implementation [44].

Within the trial, training and support for nurses and AHPs will be provided, including a discussion of potential workload concerns. The potential benefits of PIFU for patient care and clinical practice, and the similarity with many aspects of current nurse/AHP practice, will be emphasised. These include patient empowerment, PET-CT to transform patient care through identification of potential recurrence and potentially (in the longer-term) reduced clinic numbers.

The planned Quintet Recruitment Intervention [70] will collect thorough site-specific data on the recruitment process and any barriers, including selective recruitment by clinicians. Clinicians/recruiters will be encouraged to discuss concerns about the patient's ability to undertake PIFU with the patient (and any family members) prior to any decisions about trial inclusion, but will exclude patients who are not suitable for PIFU.

### Strengths and Limitations

We achieved a diverse sample in terms of clinical role and location (representing most areas of England, as well as Scotland and Wales), aided by conducting focus groups online. Some of the surgeons had already had discussions with colleagues about PETNECK2 and its rationale as part of the grant-writing process – other clinicians may be less supportive and have more concerns. Many were colleagues of PETNECK2 chief investigators, as the HNC profession is a relatively small community, which may have influenced their responses.

## Conclusion

HNC clinicians support the development and evaluation of PIFU as an alternative to inflexible and possibly ineffective current follow-up. However, barriers to help-seeking may be an issue, particularly for those already disengaged from care. PIFU will need to emphasise patient engagement and reassurance, psychosocial issues, education on symptom reporting and reliable quick routes back to clinic. Concerns about patient fear of recurrence and anxiety may be outweighed by improved fear normalisation, self-management and holistic treatment. Early, meaningful and ongoing staff engagement around the trial rationale and recruitment process is essential to discourage selective recruitment and address risk-averse behaviour and potential workload burden.

## Conflicts of interest

The authors declare no conflicts of interest.

## Funding

This work was supported by the 10.13039/501100000272National Institute for Health Research (10.13039/501100000272NIHR) under its Programme Grants for Applied Research Programme (NIHR200861). The views expressed are those of the author(s) and not necessarily those of the NIHR or the Department of Health and Social Care.
